# Avoidance of biological contaminants through sight, smell and touch in chimpanzees

**DOI:** 10.1098/rsos.170968

**Published:** 2017-11-08

**Authors:** Cecile Sarabian, Barthelemy Ngoubangoye, Andrew J. J. MacIntosh

**Affiliations:** 1 Primate Research Institute, Kyoto University, Inuyama 484-8506, Japan; 2 Centre de Primatologie, Centre International de Recherches Médicales de Franceville, Franceville BP 769, Gabon

**Keywords:** *Pan troglodytes*, parasite avoidance, bodily products, disgust, sensory modalities

## Abstract

Avoiding biological contaminants is a well-known manifestation of the adaptive system of disgust. In theory, animals evolved with such a system to prevent pathogen and parasite infection. Bodily products are human-universal disgust elicitors, but whether they also elicit avoidance behaviour in non-human primates has yet to be tested. Here, we report experimental evidence that potential exposure to biological contaminants (faeces, blood, semen), as perceived via multiple sensory modalities (visual, olfactory, tactile), might influence feeding decisions in chimpanzees (*Pan troglodytes troglodytes*)—our closest phylogenetic relatives. Although somewhat mixed, our results do show increased latencies to feed, tendencies to maintain greater distances from contaminants and/or outright refusals to consume food in test versus control conditions. Overall, these findings are consistent with the parasite avoidance theory of disgust, although the presence of biological contaminants did not preclude feeding entirely. The avoidance behaviours observed hint at the origins of disgust in humans, and further comparative research is now needed.

## 1. Introduction

Many major infectious diseases (e.g. infectious intestinal diseases, herpes, hepatitis) are transmitted via contact or ingestion of particles of bodily products such as faeces, blood, semen and saliva. The same bodily products are also known to be human-universal disgust elicitors [1]. From an evolutionary perspective, organisms are expected to have evolved diverse defence mechanisms to afford survival and reproduction while minimizing fitness losses caused by pathogens. These mechanisms are grouped under the umbrella of avoidance, resistance and tolerance, or the ART of pathogen defence (see review in [2]). Avoidance is expressed as the actions taken by an animal to reduce its probability of becoming infected, while resistance (i.e. the ability to limit the parasite/pathogen burden via the immune system) and tolerance (i.e. the ability to live with a parasite/pathogen by limiting its harmful effects) take place once infection has occurred. As such, avoidance behaviour provides animals a front-line defence against harmful parasites and pathogens.

For a given host to trigger any mechanism of avoidance, it must first detect some cue indicating infection risk. The processing of such cues in humans involves disease-relevant emotional and cognitive responses that stimulate disease-avoidance behaviours. Such cognitive responses underpin hypotheses linked to the behavioural immune system [3] and the parasite avoidance theory of disgust [1,4,5], and even form the core of Darwin's [6] definition of disgust as ‘a sensation … referring to something revolting, primarily in relation to the sense of taste, as actually perceived or vividly imagined; and secondarily to anything, which causes a similar feeling, through the sense of smell, touch and even eyesight’. Disgust, then, should be viewed here not in the traditional sense of subjective experience but instead as a system that evolved to detect signs of pathogens and other infectious agents, as well as to stimulate the expression of behaviours that reduce the risk of their acquisition [1,7,8].

In nature, infection avoidance can take different forms depending on the epidemiology of the parasite or pathogen in question. Known forms include directly avoiding or removing parasites or pathogens themselves, avoiding conspecifics with signs of infection, or avoiding contaminants or contaminated areas [7]. Specific examples include social lobsters (*Panulirus argus*) avoiding dens containing conspecifics infected with PaV1 virus and preferentially sharing dens with uninfected lobsters [9], and mandrills (*Mandrillus sphinx*) grooming heavily parasitized group members less often [10]. Other examples include *Caenorhabditis elegans* avoiding agar plate lawns containing pathogenic bacteria [11], Japanese macaques (*Macaca fuscata*) avoiding low-calorie food contaminated with fresh faeces [12], birds spending large amounts of time preening to remove ectoparasites [13] and ungulates preferentially grazing in pasture with lower faecal contamination [14–16]. However, for most species, infection risk assessment remains poorly investigated, as does the question of which sensory stimuli elicit avoidance. What we do know mainly comes from studies of invertebrates. For example, *C. elegans* can detect pathogenic bacteria via chemosensation of bacterial secondary metabolites, which then modulates neuroendocrine signalling and promotes avoidance behaviour [17]. *Drosophila melanogaster* and dung beetles (*Scarabaeus lamarcki*) can detect volatile phenols emitted by pathogenic bacteria in carnivore faeces via olfaction [18], and *D*. *melanogaster* can also detect Gram-negative bacteria through chemosensory cues of the lipopolysaccharides they produce [19,20]. Similarly, lobster avoidance of diseased conspecifics is likely to be chemically mediated, as olfaction already mediates dominance hierarchies, mate choice, foraging and aggregation in such species (see [9]). In comparison, mammalian sensory and neural systems involved in avoidance behaviours remain largely unknown. However, rodents rely mainly on olfactory cues involving the vomeronasal system [21] to discriminate between healthy and parasitized conspecifics [22,23], while ungulates seem to rely on both olfactory [24,25] and visual cues [14–16] to avoid faecal contamination.

Apart from humans, rodents and ungulates, the literature focusing on the avoidance of contaminants (i.e. bodily products) is sparse. Among non-human primates, the most relevant taxa with which to explore the origins of avoidance behaviour in humans, evidence of infection avoidance behaviour remains anecdotal or insufficient. Reported observations of potential infection risk avoidance behaviours include chimpanzees using leaves to wipe body parts soiled with faeces, sticky food, blood, urine, mud and semen [26,27], and Japanese macaques washing food with sea and fresh water [28,29]. Such examples provide the first insights into contaminant-avoidance behaviours in non-human primates, but studies experimentally testing contaminant avoidance are needed to deepen our understanding of the behavioural immune system in our closest phylogenetic relatives. Allritz *et al*. [30] showed that captive chimpanzees, bonobos and orangutans washed sand-covered apples more often than clean and peeled apples, yet did not address the potential hygienic function of such behaviour. Going a step further, we previously showed that hygienic tendencies towards faeces avoidance and food manipulation prior to ingestion correlated negatively with geohelminth infection in Japanese macaques [12]. However, only a few contaminants were tested in the latter study (i.e. faeces, sand and soil). Although Japanese macaques seemed to use visual information to avoid feeding on faeces, experiments have not yet been conducted to investigate which types of stimuli elicit avoidance behaviour in non-human primates, and what senses are involved in their detection.

**Figure 1.**
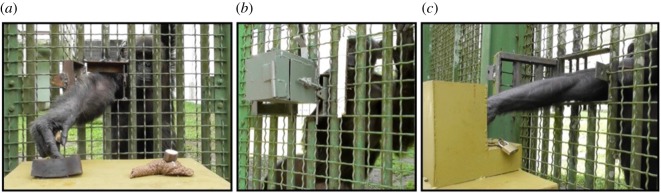
From left to right: experimental setting to test (*a*) vision-mediated avoidance of faeces (condition 1: foam control on the left and brown faeces replica on the right); (*b)* olfaction-mediated avoidance of biological contaminants; and (*c*) touch-mediated avoidance of biological contaminants.

**Figure 2.**
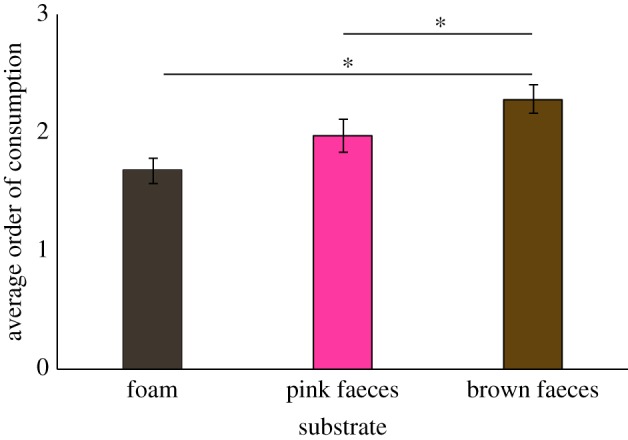
Average order of consumption for each substrate when presented simultaneously in the second condition in vision-mediated avoidance of faeces tests: grey–brown control foam (1.7 ± 0.1); pink faeces replica (2 ± 0.1); and brown faeces replica (2.3 ± 0.1). Column heights represent the mean; error bars reflect standard error of the mean; and stars reflect significant difference between the proportion of ‘take first’ from the foam and brown faeces replica, and from the foam and pink faeces replica (both *p* < 0.05).

Thus, we performed three experiments to test whether chimpanzees (*Pan troglodytes troglodytes*) are sensitive to visual, olfactory and tactile stimuli of potential contaminants and therefore: (1) recognize and avoid consuming items associated with visual stimuli of faeces (figure 1*a*); (2) avoid consuming items in a location associated with olfactory stimuli of faeces, blood or semen (figure 1*b*); and (3) avoid consuming items associated with tactile stimuli of a potentially contaminated substrate (figure 1*c*). We predicted that individuals would be more cautious regarding food associated with contaminant-like stimuli compared to control stimuli, manifest as lower probabilities to feed and higher avoidance of contaminated areas.

## 2. Results and discussion

### 2.1. Vision-mediated avoidance of faeces

In the first set of experiments, 20 chimpanzees at the Centre International de Recherches Médicales de Franceville (CIRMF) in southern Gabon were given access to bananas placed atop different substrates. In the first condition, we used two substrates, a brown faeces replica and a piece of foam used as a control. In the second condition, subjects were presented with a similar choice but an additional substrate was added, a pink faeces replica. Contrary to our predictions, in neither condition did chimpanzees consume bananas less often from the brown faeces replica than from the control foam (condition 1: 89% versus 91% respectively; see electronic supplementary material, video 1), indicating little sensitivity to contamination risk when consuming food associated with a visual representation of a potentially contaminated substrate. Similarly, there were no differences in their decisions to consume bananas atop the brown faeces replica, the control foam, and the pink faeces replica in condition 2 (90% of bananas were consumed from the brown faeces replica, and 92.5% from the pink faeces replica and the control foam). However, subjects did select the banana atop the control foam (GLMM, *p* = 0.029; figure 2, table 1) and the pink faeces replica (GLMM, *p* = 0.049; figure 2, table 1) first significantly more often than the banana atop the brown faeces replica, which they tended to consume last. These results might suggest a preference for food that is not associated with contamination risk, although it does not suggest avoidance of such risk altogether.

**Table 1.** Factors affecting variation in avoidance of visual stimuli of faeces. Bold text denotes predictor variables causing significant variation in the response.

statistical model	predictor variable	est.	s.e.	stat.	*p-*value
likelihood of feeding on visual faeces replica	(intercept)	−0.256	4.932	−0.052	0.959
	sex (male versus female)	−1.546	2.325	−0.665	0.506
	rank (low versus high)	−2.276	2.329	−0.977	0.329
	age	0.246	0.145	1.691	0.091
	trial	0.063	1.057	0.060	0.952
	audience (present versus absent)	0.395	1.402	0.282	0.778
	item (brown faeces replica versus control)	−0.756	1.264	−0.598	0.550
	item (pink faeces replica versus control)	−1.21 × 10^−5^	1.342	0.000	1.000
	item (brown faeces replica versus pink faeces replica)	−0.756	1.264	−0.598	0.550
likelihood of feeding first on visual faeces replica	(intercept)	−0.955	1.258	−0.759	0.448
	sex (male versus female)	−0.005	0.457	−0.011	0.992
	rank (low versus high)	−0.125	0.411	−0.305	0.760
	age	0.021	0.029	0.716	0.474
	trial	−0.006	0.405	−0.014	0.989
	audience (present versus absent)	−0.065	0.454	−0.144	0.885
	**item (brown faeces replica versus control)**	−1.152	0.528	−2.181	0.029*
	**item (brown faeces replica versus pink faeces replica)**	−1.046	0.531	−1.971	0.049*
	item (pink faeces replica versus control)	−0.106	0.461	−0.230	0.818

Significant *p*-values are marked: *p < 0.05.

Chimpanzees, like humans, have trichromatic colour vision, allowing them to discriminate easily and accurately between pink and brown colours. As both faeces replicas had exactly the same size and shape, and both were made from the same material (papier-mâché), these feeding preferences might derive from colour-based visual discrimination. When presented with two objects made from the same material and with similar colours but different shapes, humans [31] and Japanese macaques [12] exercised greater caution towards the substrate with faeces shape. Here, our results imply that colour may be an important factor in faeces discrimination among chimpanzees as well. The discordant (with expectations) colour of the pink faeces replica might have led chimpanzees to relax their sensitivity to other visual cues such as size and shape. However, even though chimpanzees may prefer feeding on items associated with substrates that less resemble real faeces, they did not avoid consuming bananas placed on the most realistic faeces replica.

Among Japanese macaques, there seems to be a trade-off between the nutritional value of the food and infection risk, with individuals being less reluctant to feed on faeces when grains of wheat are replaced with peanuts [12]. So, perhaps the nutritive value of the food reward used here (i.e. pieces of banana) was high enough to elicit consumption regardless of perceived contamination risk, despite the daily provisioning of bananas and the small amount given in the experiment. Another potential explanation is that tests were conducted in the morning, before feeding, meaning that chimpanzees would be rather motivated to feed during experiments. In humans, there also seems to be a trade-off between disgust and hunger, with food-deprived subjects expressing a weaker rise of the upper lip while watching pictures of unpalatable food [32]. Alternatively, it may be that chimpanzees simply recognized that the faeces-like substrates were in fact not faeces, and, therefore, exhibited little reluctance to feed. In experiments with Japanese macaques, subjects displayed intermediate levels of reluctance to feed on the replica faeces when compared to real faeces and a piece of brown plastic [12]. Together with the current results, such findings suggest that primates in general may be able to discriminate between real and replica faeces when other relevant sensory modalities are not activated, e.g. via faecal odour. Finally, it may also be that faeces do not deter chimpanzees from foraging, or at least those housed at the CIRMF.

### 2.2. Olfaction-mediated avoidance of biological contaminants

**Figure 3.**
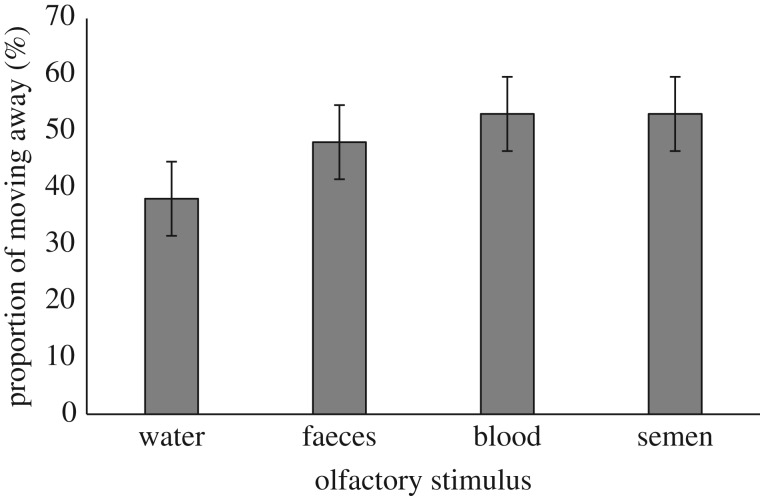
Proportion of subjects moving away from the experimental area before consumption as a function of the olfactory stimulus presented (water: 38 ± 6.6%; faeces: 52 ± 6.5%; blood: 53 ± 6.5%; semen: 53 ± 6.5%). Grey bars represent the proportion of trials during which subjects moved away from the experimental area before accessing the food reward or trial termination. Error bars represent standard error of the proportion.

In a second experiment, the same 20 chimpanzees were exposed to different olfactory stimuli of various contaminants (conspecific faeces, blood and semen). We first examined chimpanzee feeding behaviour while exposed to the different odours and found that their proportion of feeding did not differ significantly while exposed to the odours of blood (bananas consumed in 93% of trials; GLMM, *p* = 0.997) and semen (92%; GLMM, *p* = 0.649) when compared to a water control (93%). However, when food was associated with faeces odour, subjects were marginally less likely to feed than they were during control trials, despite still consuming the bananas in 83% of trials (GLMM, *p* = 0.055). We further examined chimpanzee tolerance towards these odours, observing weak tendencies for subjects to move away from the experimental area during trials and before accessing the food more often when presented with odours of blood (GLMM, *p* = 0.075; figure 3, table 2) and semen (GLMM, *p* = 0.077; figure 3, table 2) when compared to the control. Figure 3 illustrates that subjects also seemed to leave the experimental area more often before accessing the bananas associated with faeces odour than when the banana was presented with the control, although this did not receive statistical support in our models (table 2). Chimpanzees thus appeared capable of perceiving and discriminating between olfactory stimuli of potential contaminants, tending to move away from and/or consume food items less often when associated with odours of these potential contaminants. However, because in all cases such avoidance responses were weak, the threat levels perceived in these experiments may not have been great.

**Table 2.** Models for variation in avoidance of olfactory stimuli of biological contaminants.

statistical model	predictor variable	est.	s.e.	stat.	*p-*value
likelihood of feeding in an area containing olfactory stimuli of biological contaminants	(intercept)	4.583	3.495	1.311	0.190
	sex (male versus female)	−1.124	1.370	−0.820	0.412
	rank (low versus high)	−0.937	1.213	−0.772	0.440
	age	0.031	0.079	0.396	0.692
	trial	−0.006	0.339	−0.017	0.986
	audience (present versus absent)	−0.397	0.723	−0.550	0.583
	odour (blood versus control)	0.004	0.846	0.005	0.996
	odour (faeces versus control)	−1.421	0.742	−1.915	0.054
	odour (semen versus control)	−0.370	0.814	−0.454	0.650
likelihood of moving away from an area containing olfactory stimuli of biological contaminants	(intercept)	0.461	1.531	0.301	0.763
	sex (male versus female)	−0.429	0.611	−0.701	0.483
	rank (low versus high)	0.332	0.556	0.598	0.550
	age	−0.038	0.037	−1.020	0.308
	trial	−0.055	0.178	−0.310	0.757
	audience (present versus absent)	0.409	0.352	1.164	0.245
	odour (blood versus control)	0.743	0.417	1.781	0.075
	odour (faeces versus control)	0.505	0.412	1.226	0.220
	odour (semen versus control)	0.736	0.416	1.770	0.077

Chimpanzees are known to possess an olfactory sensitivity similar to that of humans, as the number of functional olfactory receptor (OR) genes does not vary significantly between the two species, but they do have a different repertory of OR genes [33]. The past decade of physiological, behavioural, anatomical and genetic research on primate olfaction has begun to shed light on the processes involved in food acquisition [34]. Evidence now suggests that olfaction may be involved in the avoidance of infected conspecifics among certain primate species [10], though how widespread this phenomenon might be, remains unknown. Previous work suggests that Japanese macaques do rely on visual stimuli in the context of faeces avoidance, as faeces replicas were sufficient to elicit some degree of avoidance behaviour, though not as strongly as real faeces [12]. Here, olfactory stimuli such as blood and semen also seemed to elicit some aversion from chimpanzees, albeit to a lesser extent. In other experiments, female Holtzman rats (*Rattus norvegicus*) distanced themselves more quickly from a glass sheet treated with the blood of conspecifics than a glass sheet treated with distilled water [35]. Domestic chicks (*Gallus gallus domesticus*) were also shown to avoid the blood of conspecifics, as well as that of heterospecifics (mice), seemingly incorporating olfaction because red dye was significantly less aversive than was blood [36]. In our study with captive chimpanzees, but also perhaps in the wild, it is possible that common exposure to odours of faeces, blood and semen may in part explain the weak avoidance of these potential contaminants observed in our experiments. Alternatively, because olfactory cues of faecal contamination elicited the strongest feeding inhibition in our subjects, perhaps the magnitude of the odour may play a role, as the odours of blood and semen may be less readily detectable or inhibitory to chimpanzee feeding behaviour.

### 2.3. Touch-mediated avoidance of biological contaminants

**Figure 4.**
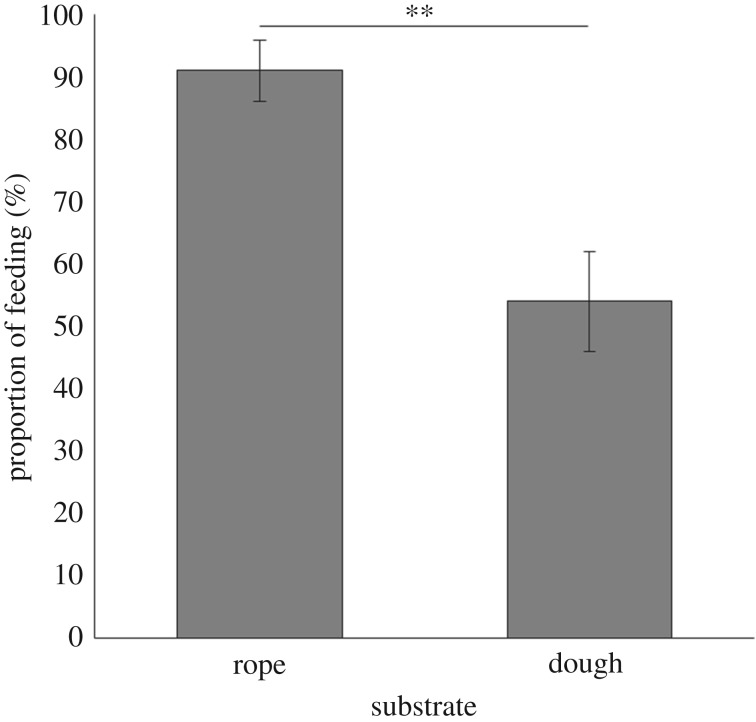
Proportion of subjects consuming food associated with either a rope substrate (91 ± 4.9%), used as a control, or a dough substrate (54 ± 8%), which mimicked the consistency, temperature and moisture of a potentially contaminated substrate. Bars represent the proportion of trials during which subjects consumed the food reward; error bars reflect standard error of the proportion; and stars reflect significance difference between the two proportions of feeding (*p* < 0.01).

In a third experiment, we presented 42 chimpanzee subjects with a foraging task while simultaneously exposing them to two hidden substrates: (1) dough, which has been used in human experiments to replicate the consistency, temperature and moisture of a potentially contaminated substrate [37], and (2) a piece of rope used as a control. Both substrates were invisible to the chimpanzees, hidden inside a box. In full view of the subjects, we placed pieces of banana in each box atop the substrate. After initiating the trials by placing their hands inside the boxes in search of the food and thereby touching the substrates, chimpanzees were significantly less likely to consume banana pieces placed on dough than those placed on rope (GLMM, *p* = 0.005; figure 4, table 3, see electronic supplementary material, video 2).

**Table 3.** Factors affecting variation in avoidance of tactile stimulus of biological contaminants. Bold text denotes predictor variables causing significant variation in the response.

statistical model	predictor variable	est.	s.e.	stat.	*p-*value
likelihood of feeding on a soft and moist substrate	(intercept)	3.552	1.99	1.782	0.075
	sex (male versus female)	0.471	0.798	0.590	0.555
	rank (low versus high)	0.512	0.801	0.639	0.523
	age	−0.003	0.037	−0.073	0.942
	audience (present versus absent)	−1.569	1.348	−1.163	0.245
	**substrate (dough versus control)**	−2.383	0.858	−2.779	0.005**

Significant *p*-values are marked: **p < 0.01.

This experiment was designed to replicate a study conducted with humans, investigating whether tactile cues play a role in the disgust response and contaminant detection/discrimination [37]. Human participants were asked to rate different substrates that varied in moisture, temperature and consistency after touching them. Wet and smooth substrates (e.g. dough) were rated more disgusting than dry and hard substrates (e.g. rope). When presented with a feeding decision, chimpanzees exhibited a pattern consistent with the results of these human experiments, demonstrating an aversion to the soft and moist substrate. Such results support the hypothesis that tactile cues are important in mediating contamination risk sensitivity. Many pathogenic organisms thrive in dark, warm and moist environments that facilitate their growth and development [38]. However, tactile perception is often overlooked in studies of pathogen avoidance. It is thus interesting that, across the three experiments, the one testing for tactile effects gave the strongest result. Whereas visual and olfactory information may allow for preventive measures to be taken to avoid contact with biological contaminants, tactile information can only be gained once contact has been made, and subjects' responses are thus reactive to such contact. This difference may underlie the stronger response observed in our tactile experiments, though further experiments are required before we can conclude that tactile information may generally lead to stronger aversion. Finally, we cannot rule out a potential effect of novelty or at least less exposure to substrates with the qualities of dough, as opposed to rope, which may have contributed to a ‘surprise’ effect in the tactile experiments. Nonetheless, this result is consistent with our expectations if chimpanzees are primed to avoid or react strongly to substrates that may pose a risk of infection.

In our study, only adult chimpanzees were tested and we did not observe any significant influence of age, dominance rank, sex, habituation (i.e. trial) or audience on feeding decisions, feeding preferences or avoidance responses to visual, olfactory or tactile cues of potential contaminants. By avoiding bodily products of unknown individuals, chimpanzees can benefit from avoiding a potentially large array of parasites and pathogens: e.g. *Salmonella typhi*, *Escherichia coli*, *Shigella*, *Rotavirus*, *Giardia* in faeces; acquired immune deficiency syndrome, cytomegalovirus (CMV) infection, hepatitis B, C and D in blood; and chancroid, chlamydial infection, herpes, CMV in semen. Regarding pathogen transmission via faecal material, it is important to note that coprophagy, which is commonly observed in wild and captive chimpanzees, mainly targets the reingestion of seeds from an individual's own faeces [39,40], which is less risky in terms of novel pathogen acquisition than consuming the faeces of conspecifics. Furthermore, consuming items excreted in faeces may reflect the trade-off dependent upon the potential costs and benefits of engaging in such risky behaviour, as noted above and in our study with Japanese macaques [12]. Such a system of override is not incompatible with a general aversion to faeces and other biological contaminants, so further research should aim to determine under which conditions chimpanzees and indeed other animals engage in risk-prone and risk-averse behaviours.

Taken together, our results suggest that the adaptive system of disgust might play a role in mediating feeding decisions of chimpanzees, and that these primates incorporate multiple sensory modalities in so doing, although it does not preclude their engaging in potentially risky foraging behaviours from the perspective of pathogen acquisition. If chimpanzees and other primates can discern contamination risk via multiple modalities, individuals with higher sensitivities to potential biological contaminants may express lower rates of infection [12], which could have important health and fitness benefits. Though again, this would depend on the nature of the resource and contaminant encountered, and may vary both across individuals and across contexts. Considering captive animals, our results might also have implications for animal welfare and management, e.g. by better informing staff and keepers about the adaptive value of such sensitivity and its flexibility, as well as identifying specific individuals that may be more prone to infection and thus require more health-related interventions. Being our closest phylogenetic relatives, chimpanzees are a good model with which to study the origins of human disgust. We, therefore, encourage future research on this but also other species, to investigate the role of learning in pathogen avoidance, the physiology of the adaptive system of disgust and the cognitive processes underlying pathogen avoidance.

## 3. Material and methods

### 3.1. Study site and subjects

All experiments were conducted between September and December 2015 at the CIRMF in southern Gabon. We tested 41 adult chimpanzees (21 females, 20 males; age ± s.d. = 27.0 ± 9.6 years) from six groups housed separately in indoor buildings (19 × 7 m) connected in pairs to three outdoor enriched courtyards (22 × 43 m) (see electronic supplementary material, table S4, for details about individual subjects). A sanctuary for the retirement of those chimpanzees is now under construction on islands in southwest Gabon. To facilitate medical interventions and biological sample collection, subjects could be isolated in raised single cages connected to their indoor buildings. These chimpanzees have not been involved in any biomedical research protocols since 2002. They are fed twice daily with seasonal fruits and vegetables, as well as with a mixture of baked soya beans and wheat flour.

### 3.2. Experimental procedures

All experiments were conducted in the morning, before feeding, at the cage of the three courtyards (figure 1) that connect to their respective buildings (B1–B6) via a metallic sliding door allowing separation. Chimpanzees performed the tests via small openings on the cage that allowed them to pass an arm through and reach food (figure 1). All subjects were tested individually, either while fully isolated from the rest of the group via the indoor/outdoor separation (as often as was possible: 68% of 453 tests), or partially isolated with one or several other members of the group in the courtyard being distracted with food by a keeper at a location well away from the experimental area. Subjects were never presented with the same test conditions on the same day. All experiments were conducted in a feeding-context, and attempted to simulate infection risk by presenting subjects with models or odours of potential contaminants while minimizing their risk of actually acquiring a pathogen from the biological products of conspecifics. All experiments were recorded with a Panasonic HC-W570M video camera mounted on a tripod, placed 2 m away from the experimental area. All experimental procedures were approved by the Animal Welfare and Animal Care Committee of the Kyoto University Primate Research Institute.

### 3.3. Vision-mediated avoidance of faeces

In the first experiment, we tested 20 chimpanzees (10 females, 10 males) for faeces avoidance via visual stimuli. In the first condition, we presented subjects with a foraging task involving a piece of banana placed atop each of two substrates: a piece of brown painted foam (control substrate) and a brown painted papier-mâché faeces replica (15 × 5 cm; Sanromà I, S. L.; Barcelona, Spain; REF. 21D). Substrates were paired and placed side-by-side on a painted metallic table (50 × 30 × 75 cm). In the second condition, we presented subjects with the same two items but added a pink-coloured faeces replica with the same dimensions and made from the same material as the original. The three substrates were aligned 15 cm apart on the table. All substrates were fixed on the table with a screw. Once the subject was isolated, we called him/her to the experimental area and moved the table forward to within an arm's reach. The test was initiated once the subject came to within 1 m of the experimental area, and was terminated when the bananas were ingested, the subject moved further than 2 m away from the experimental area, or after 150 s if neither of these behaviours occurred. Each subject was given five trials in condition 1 (*N* = 100 tests) and two trials in condition 2 (*N* = 40 tests). Tests in condition 2 were conducted after the five trials in condition 1 had been completed, as a means of testing for discrimination between visual components, i.e. colour versus size and shape. We alternated the position (side) of each substrate across trials for each subject to avoid the possible influence of side biases.

### 3.4. Olfaction-mediated avoidance of biological contaminants

In a second set of experiments, we tested the same subjects on the olfaction-mediated avoidance of faeces, blood and semen. Volatile compounds present in organic material allowed us to test a wider range of contaminants in this experiment, based on olfactory stimuli, compared to the two other experiments. We fixed a metallic feeding box (20 × 12 × 16 cm) on the grid of the indoor enclosure, and then attached a 25 cm piece of bamboo vertically along the grid, approximately 10 cm from the feeding box. We then coated the sides and back of the bamboo with one of the above-mentioned contaminants, or with a water control; applying roughly the same quantity of contaminant/control material to the bamboo across trials with a spray bottle or spatula (i.e. approx. 0.5 ml for blood, 1.5 ml for semen and 3 g for faeces). The box was closed in the front with a vertical sliding door and the food was placed in the box via a back door (figure 1*b*). Chimpanzees were attracted towards the experimental area with a piece of banana, presented to them just behind the bamboo with the tested olfactory stimulus for 3 s to make sure they were exposed to the contaminant odour. The piece of banana was subsequently placed inside the box. The experimenter would then step back and wait for 30 s to let the subject investigate the olfactory stimulus before opening the front door of the box, giving access to the piece of banana. Subjects were not in visual contact with the recipient containing the contaminant/control. We alternated the side of the piece of bamboo to the box across trials for each subject. Each trial lasted for 150 s, starting when the subject approached to within 1 m of the experimental area. Each subject was tested three times for each olfactory stimulus (*N* = 60 tests/olfactory stimulus; *N* = 240 tests in total). A new sample of the same olfactory stimulus was added to the bamboo between each test for consistency.

Faecal samples presented to subjects of one group came from chimpanzees of another group and were collected from different donors. Blood samples were collected during nine health checks on eight different healthy chimpanzees not coming from any of the six tested groups. Semen samples came from 23 separate ejaculates of one chimpanzee donor infected with the simian immunodeficiency virus, and were collected after the individual masturbated and spread his semen on the bars of his enclosure. This individual was also isolated from the six test groups. Faecal samples were collected on the day of the experiments and kept in a cooler box (without ice) until experiments were performed. Blood and semen samples were collected and immediately placed in a freezer (−20°C) until the day of the experiments, a few hours before which the required quantity was removed from the freezer and thawed.

### 3.5. Touch-mediated avoidance of biological contaminants

In a third experiment, we tested 41 chimpanzees of the same six groups, replicating a study conducted in humans investigating the role of touch in eliciting avoidance of substrates that model those associated with contamination risk [37]. We welded a metallic box (figure 1*c*) with a false bottom to a table like that used in the first experiment. The target subject was solicited to a table at the experimental area with the opening in the enclosure grid closed. The experimenter showed a piece of banana to the subject and then placed it in the false bottom of the box, where either a piece of rope or dough (16 × 6 cm) was placed beforehand. As such, the piece of banana lay on one of the two invisible (to the subjects) substrates, and the configuration of the box made it impossible for the chimpanzees not to touch the substrate when attempting to extract the banana. The experiment was initiated when the target subject was within 1 m of the opening and the experimenter unlocked the opening in the grid, allowing the subject to reach inside the box. The dough recipe was the same as that used in the human study we were replicating [37], and was designed to present a soft, moist substrate to the subjects. After preparation, dough was kept in a cooler box (without ice) until experimentation, to protect it from the elements and to preserve it from decomposing too quickly. Each subject was tested only once in each condition to prevent them from removing the dough out of the box and being exposed to visual stimuli, hence more subjects were tested than in the two previous experiments. On the 41 subjects who entered the experimental area (within 2 m of the apparatus), 34 chimpanzees reached inside the box in the rope condition, and 39 in the dough condition. Analyses of subject responses are based only on this subset of the data in which subjects participated actively in the experiments. The test was terminated when the subject ingested the banana, moved more than 2 m away from the experimental area, or after 150 s.

### 3.6. Data analysis

We built generalized linear mixed-effects models (GLMM) to analyse the responses of subjects in the three experiments. For the first experiment, response variables included first whether the banana was consumed or not (feeding decision) and second whether the banana was consumed first or not (feeding preference) on each substrate used in the tests. In both models, constructed using a binomial error structure with logit link function, predictor variables included the experimental substrate (control foam, brown faeces replica, pink faeces replica), the age, dominance rank and sex of each subject, trial number (to test for habituation/sensitization effects) and an audience effect (i.e. the presence or absence of other individuals in the courtyard). For the second experiment, the first set of models included feeding decision (consume or not) across trials as a binary response. A second set of models tested for direct olfactory stimulus avoidance, setting whether or not the subject moved more than 2 m away from the experimental area before opening the box as a binary response. For both sets of models, olfactory stimulus (faeces, blood, semen or water), age, dominance rank, sex, trial number and audience were used as predictor variables. In each of the above models, we controlled for individual identity and trial to account for pseudoreplication by including them as random effects. Furthermore, each subject's identity was nested within their respective groups to account for group-level variation. For the third experiment, we constructed a single model with feeding decision as a binary response variable, with test substrate (rope or dough) and subject age, dominance rank and sex, as well as audience used as predictor variables. Individual identity was again used as a random effect, nested within group origin. All data were analysed in R v. 3.3.3.

## Supplementary Material

Participating subject details

## Supplementary Material

Sarabian data visualcond1 ESM

## Supplementary Material

Sarabian data visualcond2 ESM

## Supplementary Material

Sarabian data olfactory ESM

## Supplementary Material

Sarabian data tactile ESM

## Data accessibility

Supporting data are accessible in electronic supplementary material.
