# Supplementary material for: Avoidance of biological contaminants through sight, smell and touch in chimpanzees
Source: R Soc Open Sci. 2017 Nov 8;4(11):170968. doi: 10.1098/rsos.170968 (PMC5717664; doi:10.1098/rsos.170968)
Supplement: Participating subject details [file rsos170968supp1.docx]

Electronic Supplementary Materials

**Avoidance of biological contaminants through sight, smell and touch**

**in chimpanzees**

Cecile Sarabiana, 1, Barthelemy Ngoubangoyeb, and Andrew J. J. MacIntosha

aPrimate Research Institute, Kyoto University, Inuyama 484-8506, Japan

bCentre de Primatologie, Centre International de Recherches Médicales de Franceville,

Franceville B.P. 769, Gabon

1To whom correspondence should be addressed. Email: [sarabiancecile@gmail.com](mailto:sarabiancecile@gmail.com)

**Table S4**. Participating subject details. Sex is given as m for males and f for females, age is given in the number of years at the time of the study, and dominance rank is based on social interactions recorded by the staff during the study period. Vision, Olfaction and Touch reflect the three sensory modalities for which subjects have been tested (indicated as “x” or blank if not tested); R and D indicate whether subjects have been tested for the rope and dough conditions, respectively.

| Subject | Sex | Age | Dominance rank | Group | Vision | Olfaction | Touch |
| --- | --- | --- | --- | --- | --- | --- | --- |
| Aboume | m | 36 | high | 6 | x | x | R, D |
| Adanhe | m | 15 | low | 2 | x | x |  |
| Amelie | f | 38 | low | 2 | x | x | R, D |
| Ayrton | m | 22 | low | 5 | x | x | R, D |
| Benefice | f | 49 | low | 4 | x | x | R, D |
| Bernadette | f | 20 | low | 1 |  |  | R, D |
| Bernard | m | 33 | low | 1 |  |  | R, D |
| Boubou | m | 13 | high | 4 |  |  | R, D |
| Brigitte | f | 23 | low | 5 |  |  | R, D |
| Cabinda | m | 13 | high | 2 |  |  | R, D |
| Charles | m | 30 | high | 4 | x | x | R, D |
| Chiquita | f | 38 | low | 2 | x | x | R, D |
| Coco | m | 20 | high | 2 |  |  | D |
| Diela | f | 14 | low | 3 |  |  | D |
| Doris | f | 25 | low | 6 |  |  | R, D |
| Fifi | f | 22 | low | 4 | x | x | R, D |
| Gentil | m | 19 | high | 1 |  |  | D |
| Geraldine | f | 26 | low | 1 |  |  | D |
| Judy | f | 39 | high | 5 | x | x | R, D |
| Julie | f | 35 | low | 5 |  |  | R, D |
| Julio | m | 17 | high | 5 |  |  | R, D |
| Junior | m | 16 | high | 5 |  |  | R, D |
| Lalala | f | 36 | high | 3 | x | x | R, D |
| Leconi | m | 14 | high | 3 | x | x | R, D |
| Letili | f | 27 | low | 6 |  |  | R, D |
| Makata | m | 34 | low | 5 | x | x | R, D |
| Makoku | f | 36 | high | 2 | x | x | R, D |
| Mandjimi | f | 14 | low | 2 |  |  | R, D |
| Masuku | f | 38 | low | 2 | x | x | R, D |
| Matthias | m | 20 | high | 2 |  |  | D |
| Mebale | f | 29 | low | 4 | x | x | R, D |
| Mgbadolite | m | 32 | high | 5 | x | x | R, D |
| Moanda | m | 37 | low | 1 | x | x | R, D |
| Morphee | f | 37 | high | 4 | x | x | R, D |
| Mpassa | m | 37 | low | 1 |  |  | R, D |
| Noemie | f | 34 | low | 3 |  |  | R, D |
| Ntoum | m | 26 | low | 1 | x | x | R |
| Nyonie | f | 18 | low | 3 |  |  | D |
| Nzela | f | 18 | low | 1 |  |  | R, D |
| Pat | m | 18 | high | 4 |  |  | R, D |
| Vaillant | m | 37 | low | 4 | x | x | R, D |

**Video 1**.

Vision-mediated avoidance of faeces, condition 1. Adult male chimpanzee, is first feeding atop foam control (left) then, atop brown faeces replica (right).

**Video 2**.

Touch-mediated avoidance of biological contaminants, “dough” condition. Adult female chimpanzee, attempts to reach food inside an opaque box. Here, the food reward lies on a dough substrate. After touching the substrate, she refused to feed.
